# The immunometabolic nature of MASH: framing immune cells within metabolic dysfunction

**DOI:** 10.3389/fimmu.2026.1797949

**Published:** 2026-04-30

**Authors:** Sergio González-Serrano, Alina-Iuliana Onoiu, Andrea Jiménez-Franco, Vicente Cambra-Cortés, Jordi Camps, Jorge Joven

**Affiliations:** 1 Unitat de Recerca Biomèdica, Hospital Universitari Sant Joan, Institut de Recerca Biomèdica Catalunya Sud, Universitat Rovira i Virgili, Reus, Spain; 2 Department of Medicine and Surgery, Faculty of Medicine, Universitat Rovira i Virgili, Reus, Spain; 3 The Campus of International Excellence Southern Catalonia, Tarragona, Spain

**Keywords:** hepatic macrophages, immune dysregulation, lipid metabolism, liver inflammation, lymphocytes, obesity, oxidative stress, T cells

## Abstract

Metabolic dysfunction-associated steatohepatitis (MASH) is a major challenge in hepatology. Despite considerable progress in our understanding of MASH, the particular mechanisms underlying disease development and the variable progression among patients with similar clinical risk factors remain inadequately explained. The liver contains one of the most varied populations of resident immune cells in the body. Accumulating evidence indicates that immune activity substantially influences the clinical trajectory of hepatic diseases. Myeloid and lymphoid cell subsets respond differently to local signals, either sustaining inflammation or facilitating tissue repair and disease remission. In MASH, metabolic and immunological pathways reinforce each other, creating a self-perpetuating pathogenic circuit. The hepatic microenvironment reprograms the metabolism and function of immune cells, while aberrant immune responses intensify hepatic metabolic stress. Consequently, MASH should be recognized as an immunometabolic disorder. Immune dysregulation is not simply a secondary effect of metabolic dysfunction but a principal driver of disease progression. Therefore, immune modulation is a central therapeutic approach. Addressing the complexity of MASH requires a thorough comprehension of hepatic immune dynamics. This review examines recent findings that place immune cells at the core of MASH pathogenesis and contends that effective management should integrate metabolic and immunological perspectives.

## 1 Introduction: MASH as a multifactorial disease with escalating global burden

Before 1980, alcohol abuse was the leading cause of chronic liver disease, and the prognosis for patients was poor. At that time, some patients, often those with obesity, diabetes, or both, confronted medical skepticism by insisting on their minimal alcohol consumption. Despite their efforts, they were unsuccessful in changing medical perceptions. A small group of pioneers, after conducting several liver biopsies, identified distinctive histological patterns and described a new disease. This condition was called nonalcoholic steatohepatitis (NASH) and was considered part of a broader spectrum of liver diseases (1). Over time, the terminology surrounding this condition has evolved to better reflect a focus on metabolic dysfunction and its implications beyond the liver. To date, accepted terms are metabolic dysfunction-associated steatotic liver disease (MASLD), which encompasses the entire spectrum of steatotic hepatic disease, and metabolic dysfunction-associated steatohepatitis (MASH), which represents its inflammatory stage (2).

MASLD has become the most common chronic liver disease, affecting up to 38% of the adult population worldwide and more so among specific subgroups such as those with obesity or type 2 diabetes mellitus. The mechanisms of progression are not entirely understood. The process initiates with hepatic lipotoxicity and may advance through stages of inflammation, fibrosis, cirrhosis, hepatocellular carcinoma (HCC), and eventually liver failure requiring transplantation. The clinical course varies considerably among patients. Approximately 10% to 30% of patients with steatotic liver progress to MASH, substantially increasing the risk of life-threatening complications. These statistics emphasize the global burden of MASH on health care (3, 4).

The strong association between obesity and MASH has established a conceptual framework in which increased adiposity is considered a main driver of hepatic fat accumulation and disease (5). However, epidemiological evidence challenges this linear relationship. Up to 19% of patients are lean, and 40% are non-obese. Paradoxically, lean patients can develop aggressive steatohepatitis, while some severely obese individuals remain protected from disease progression (6, 7). Therefore, the manifestation of MASH is highly heterogeneous.

Current therapeutic strategies for MASH are based on a metabolic-centric framework, with weight reduction as the primary intervention (8). This perspective has established bariatric surgery and certain drug agents as effective treatments due to their capacity to promote weight loss. However, recent evidence challenges this paradigm: some of these interventions achieve effectiveness through pathways independent of weight loss, such as direct immunomodulatory actions (9, 10). Therefore, clarifying the dynamics and mechanisms of the immune system in MASH is necessary.

The progression from simple steatosis to MASH comprises complex bidirectional metabolic and immune interactions (11). The liver functions as a sentinel organ, uniquely positioned to orchestrate immune tolerance while maintaining capacity for strong defensive responses (12). Disruption of this immunological equilibrium precipitates diverse pathological outcomes, including MASH and hepatocellular carcinoma (13). However, current metabolic frameworks fail to adequately explain the contribution of immune dysregulation to liver disease. Disturbances in hepatic metabolic homeostasis promote the activation and metabolic reprogramming of immune cells. In turn, immune cells can exacerbate metabolic alterations in the liver (14). These reciprocal interactions underscore the immunometabolic nature of MASH. However, immune mechanisms are not limited to pathogenic inflammation but may also contribute to MASH remission. Therefore, the activity of immune cells in liver disease is very complex (15, 16). This review provides a comprehensive analysis of the hepatic immune landscape in MASH, focusing on immune cell activity under hepatic metabolic dysfunction and the reciprocal interactions between metabolic and immune dimensions.

## 2 The systemic and immunometabolic nature of MASH

### 2.1 Determinants of hepatocellular injury and the inflammatory response

The development of MASH results from the interaction of systemic and hepatic factors that promote metabolic dysfunction in the liver and trigger an inflammatory response. The initiation of this inflammatory process is complex and involves multiple factors.

**Figure 1:**
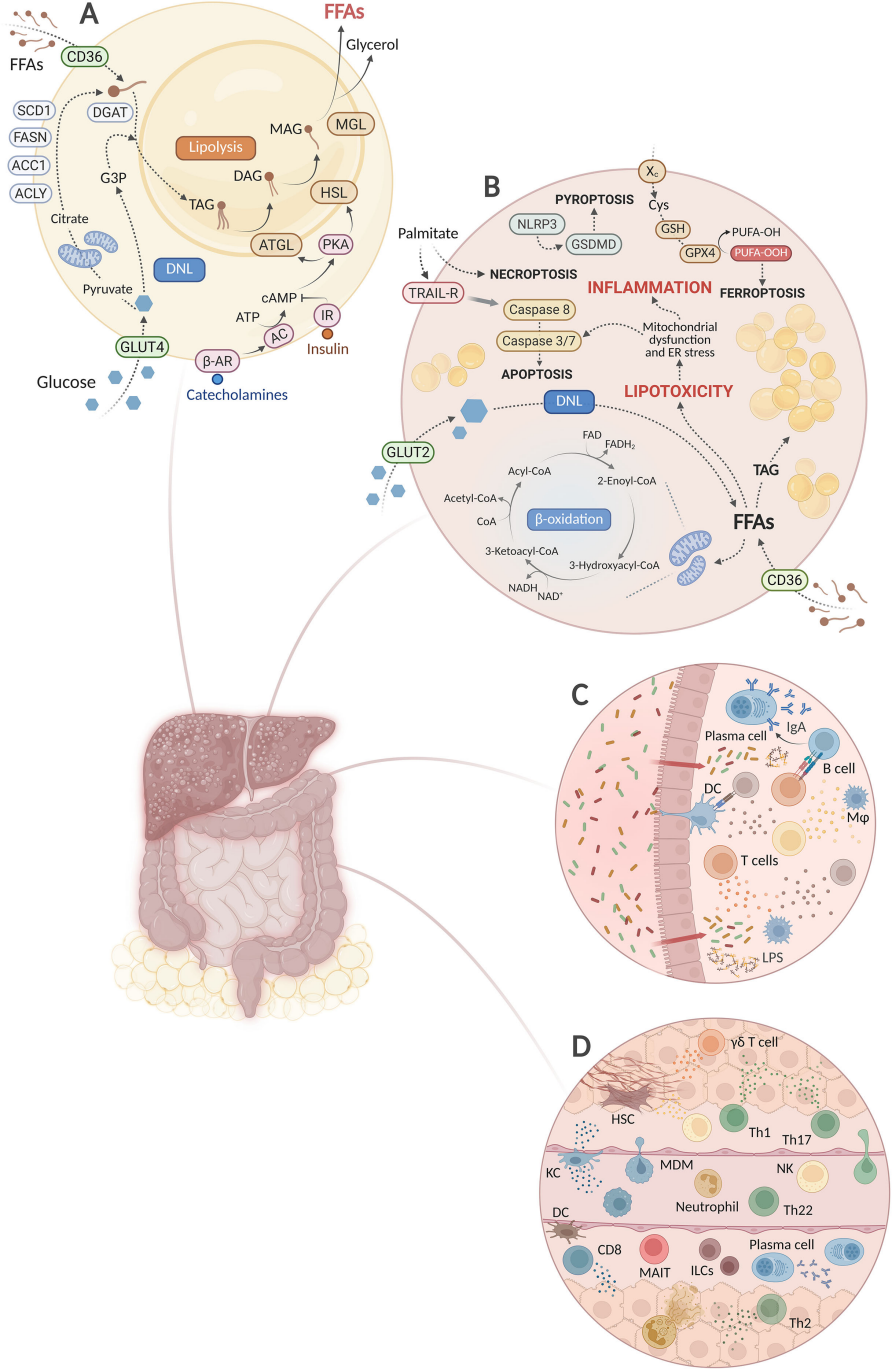
Systemic metabolic and immune dysregulation in MASH. **(A)** Chronic caloric excess results in adipose tissue lipid storage overload, increased *de novo* lipogenesis (DNL) and lipolysis, and elevated release of free fatty acids (FFAs) into the circulation. **(B)** Increased lipid influx and ectopic fat accumulation induce lipotoxicity, cell death, and inflammation in the liver. **(C)** Gut dysbiosis leads to local intestinal inflammation. Disruption of the intestinal barrier increases the translocation of microbial products into circulation, exacerbating systemic and hepatic inflammation. **(D)** Systemic and intrahepatic signals activate distinct hepatic immune cell populations, each contributing differently to disease development. Created in BioRender. Joven, J. (2026) https://BioRender.com/bxxjdet.

The adipose-liver axis is a critical initiator of pathological progression. Under chronic caloric excess, adipocytes are exposed to a sustained oversupply of fatty acids and glucose, which initially favors triglyceride synthesis and storage. Fatty acids can be directly esterified into triglycerides, while glucose contributes to *de novo* lipogenesis (DNL). As this excess persists, the storage capacity of adipose tissue is exceeded, leading to oxidative stress and immune cell infiltration (17). The subsequent secretion of cytokines, such as IL-1, IL-6, and TNF-α, establishes a systemic inflammatory state associated with insulin resistance. Under physiological conditions, lipolysis is stimulated by catecholamines via β-adrenergic signaling and suppressed by insulin. In obesity, insulin’s inhibitory effect diminishes, increasing the rate of triglyceride hydrolysis and releasing greater quantities of free fatty acids (FFAs) and glycerol into the circulation (18) (**Figure 1A**).

The overload of FFAs promotes ectopic lipid accumulation in non-adipose tissues. Hepatocytes may respond to increased FFAs influx by upregulating the lipid transporter CD36, promoting fatty acid internalization and insulin resistance (19). In parallel, excess glucose is converted into fatty acids via hepatic DNL. Mitochondria initially respond to FFAs overload by increasing β-oxidation capacity and biogenesis. However, these compensatory mechanisms are insufficient to meet the sustained metabolic demand (20). Excess FFAs are therefore converted to triglycerides, leading to hepatic steatosis (21) (**Figure 1B**). Prolonged metabolic stress impairs mitophagy and reduces mitochondrial function, as indicated by decreased oxidative phosphorylation, impaired ATP synthesis, and elevated reactive oxygen species (ROS) generation (22). The resulting oxidative burden damages cellular lipids, proteins, and mitochondrial DNA, perpetuating self-sustaining injury cycles. Furthermore, disruption of endoplasmic reticulum homeostasis activates the unfolded protein response (UPR), which is critical for regulating hepatic lipid metabolism, inflammation, and cell death pathways (23).

Hepatic lipid overload can also directly initiate inflammatory signaling by activating resident immune cells. *In vitro* studies indicate that hepatic resident macrophages detect elevated free fatty acids and respond by increasing TNF-α secretion that, in turn, promotes the progression of steatosis in hepatocytes (24). The following sections of this review will examine how metabolic alterations influence immune cell activation.

Metabolic burden in hepatocytes activates multiple cell death pathways, including apoptosis, necroptosis, pyroptosis, and ferroptosis, which can further amplify the inflammatory and fibrogenic response (**Figure 1B**). Among these, apoptosis, characterized by cell volume reduction and formation of apoptotic bodies, is considered the least inflammatory form of cell death. Palmitate can induce ligand-independent pro-apoptotic signaling through the TRAIL death receptor 5 (DR5) in hepatocytes (25). Moreover, mitochondrial dysfunction in hepatocytes promotes cytochrome c release and activation of the intrinsic apoptotic pathway (26). Pyroptosis establishes a more direct connection between hepatocyte death and inflammation. In brief, its canonical pathway is activated by damage-associated molecular patterns (DAMPs) or pathogen-associated molecular patterns (PAMPs), which stimulate the assembly of the NLRP3 inflammasome, gasdermin D pore formation, and release of IL-1β and IL-18. In primary mouse and human hepatocyte cultures, extracellular NLRP3 inflammasome particles released after pyroptosis are internalized by hepatic stellate cells, which then activate a profibrogenic response (27). Necroptosis is characterized by loss of membrane integrity and cellular swelling, leading to the release of DAMPs that further promote inflammation. While necroptosis may contribute to MASH development in both mice and humans, the specific stimuli responsible for its activation remain unclear (28). Experimental studies have linked necroptosis to palmitate exposure in murine hepatocytes and to oxidative stress in Sod1-deficient mice (29, 30). Ferroptosis is an iron-dependent form of hepatocyte death. Oxidative stress facilitates iron-catalyzed peroxidation of polyunsaturated fatty acid (PUFA) residues in membrane phospholipids, leading to the formation of lipid hydroperoxides. When antioxidant defenses, particularly glutathione peroxidase 4 (GPX4)-mediated detoxification, are compromised, these toxic lipids accumulate, resulting in membrane damage and cell death. The subsequent release of oxidized lipids and DAMPs may further contribute to hepatic inflammation (31). However, evidence regarding ferroptosis in human MASH is limited. Overall, while hepatocyte cell death may facilitate the progression from hepatic metabolic stress to inflammation, the relevance of each specific cell death pathway in human MASH has yet to be clarified.

Beyond metabolic alterations and cell death, the gut-liver axis is another important amplifier of hepatic inflammation. Gut dysbiosis, defined as an imbalance between beneficial and harmful gut microorganisms, is frequently observed in patients with obesity-associated MASH. This alteration can promote local intestinal inflammation and contribute to disruption of the intestinal barrier (32, 33) (**Figure 1C**). Increased intestinal permeability allows the translocation of harmful bacterial metabolites and antigens, such as lipopolysaccharide (LPS), from the intestinal lumen into the portal and systemic circulation. These antigens can reach the liver and trigger inflammatory responses (34).

In summary, adipose tissue dysfunction, hepatocellular metabolic stress, cell death, and gut-derived stimuli converge from the early stages of disease to activate hepatic immune cells and determine their behavior. Understanding these interactions may guide the development of therapies that target inflammation and fibrogenesis at their sources rather than addressing their consequences.

### 2.2 Hepatic immune cell composition in homeostasis and immunometabolic dysregulation in MASH

The liver contains functionally diverse resident immune cell populations. Antigen-presenting cells (APCs) modulate immunological tolerance under physiological conditions, preventing aberrant immune activation while preserving immune surveillance (35). APCs include dendritic cells (DCs), Kupffer cells (KCs), hepatic stellate cells (HSCs), and liver sinusoidal endothelial cells (LSECs). Beyond APCs, the hepatic immune landscape comprises a spectrum of specialized lymphoid populations, including tissue-resident innate-like T cells such as mucosal-associated invariant T (MAIT) cells, natural killer T (NKT) cells, and γδ T cells, as well as innate lymphoid cells (ILCs) and conventional CD4^+^ and CD8^+^ T cells. The humoral immune component includes naive B cells and antibody-producing plasma cells. Collectively, this immunological system maintains hepatic homeostasis and enables the initiation of effective defensive responses when necessary (36).

The inflammatory cascade in MASH involves diverse subsets of innate and adaptive immune cells (**Figure 1D**). To facilitate the understanding of this process, it could be broadly divided into three phases (37). Initially, the previously described pro-inflammatory stimuli are detected by hepatic resident immune cells, particularly Kupffer cells and innate-like T-cell subsets such as γδ T cells, and initiate the inflammatory response (38, 39). Then, myeloid cells, including monocyte-derived macrophages and neutrophils, are progressively recruited to the liver and amplify inflammation (40, 41). Finally, in advanced phases, the diseased liver acquires a fibrogenic architecture, reflecting both the cumulative damage from earlier stages and a more predominant adaptive immune response, mainly involving pro-fibrogenic CD4^+^ T-cell subsets and cytotoxic CD8^+^ T-cell populations (42, 43). Macrophages with reparative and tissue-remodeling functions are also found at advanced stages (44). This is, however, a simplified description, as the inflammatory process in MASH is not a fixed sequence in which distinct immune cell populations act in order. Rather, it is shaped by continuous, complex interactions among multiple immune cell populations.

The microenvironment of MASH induces metabolic reprogramming in immune cells that shape their functions. The mechanistic evidence supporting these changes derives from preclinical models in different experimental settings. For example, palmitic acid, cholesterol crystals, and lipopolysaccharide can stimulate glycolysis and lipogenesis in macrophages, while impairing the tricarboxylic acid cycle and oxidative phosphorylation (45). These changes promote macrophage polarization toward a proinflammatory phenotype. In CD8^+^ T cells, hepatic lipid accumulation impairs both glycolysis and fatty acid oxidation, reducing their cytotoxic activity (45). While hepatic metabolic dysfunction remodels immune cell behavior, activated immune cells can intensify or ameliorate hepatocyte injury and metabolic disturbances. The complex reciprocal reinforcement between metabolic and immunological pathways makes MASH an immunometabolic disease.

## 3 The role of innate immunity in MASH

### 3.1 Kupffer cell activation and monocyte recruitment

**Figure 2:**
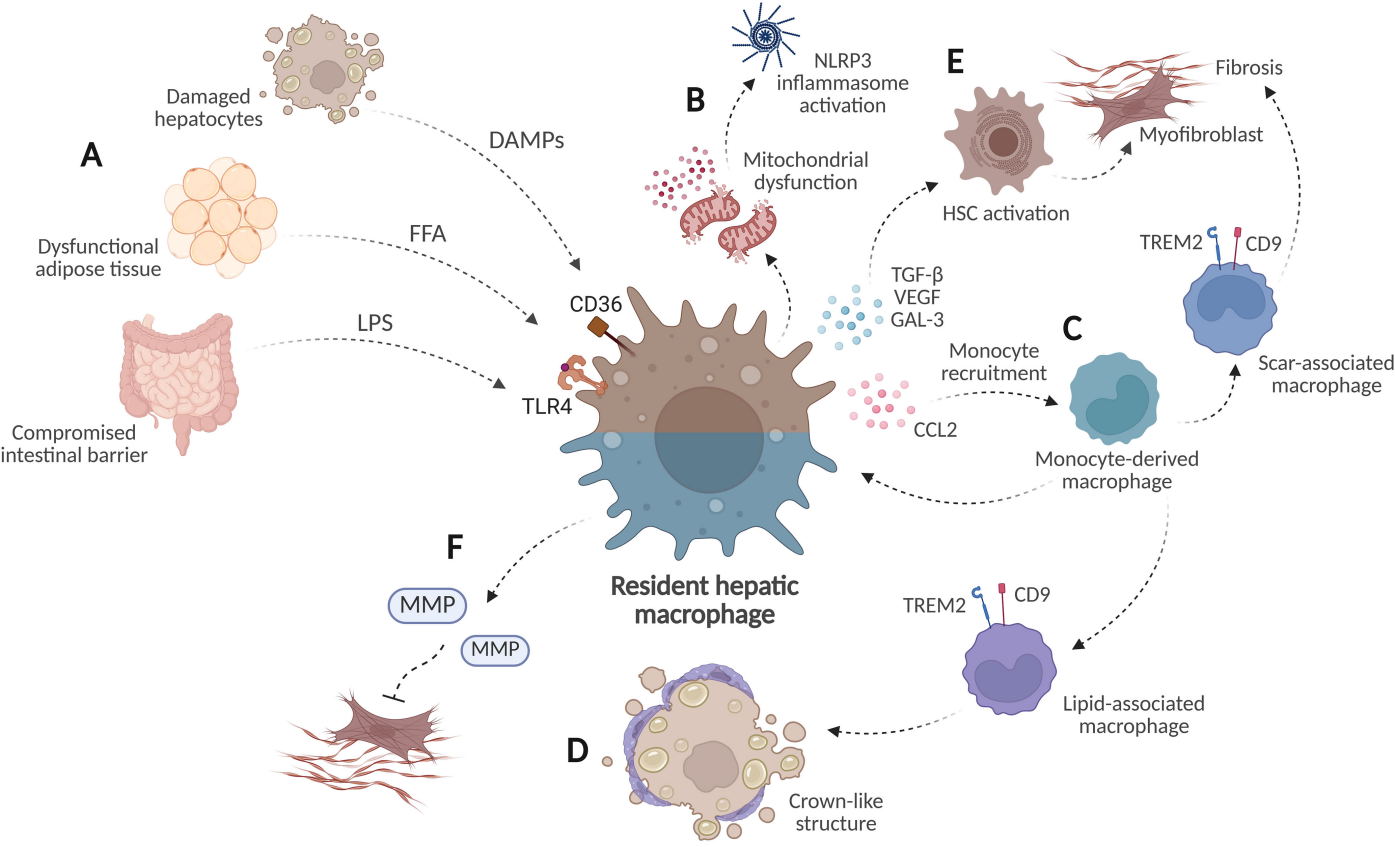
Heterogeneous functions of hepatic macrophages in MASH. **(A)** Kupffer cells are activated by gut-derived lipopolysaccharide (LPS), free fatty acids (FFAs), and damage-associated molecular patterns (DAMPs) released from injured hepatocytes. **(B)** Lipotoxic stress induces mitochondrial dysfunction and NLRP3 inflammasome activation. **(C)** Cytokines and chemokines secreted by activated Kupffer cells promote monocyte recruitment into the liver. These monocytes can differentiate into lipid-associated macrophages (LAMs) and scar-associated macrophages (SAMs). **(D)** LAMs accumulate around dying steatotic hepatocytes and lipid droplets, forming crown-like structures involved in lipid clearance. **(E)** Macrophages facilitate hepatic stellate cell (HSC) activation by releasing pro-fibrogenic mediators. SAMs accumulate in fibrotic regions and may contribute to the fibrogenic response. **(F)** After hepatic insult cessation, macrophages may promote fibrosis regression by releasing matrix metalloproteinases (MMPs). Created in BioRender. Joven, J. (2026) https://BioRender.com/kuvcrx7.

Kupffer cells are the predominant hepatic macrophage population in the healthy liver and are strategically located within sinusoids to facilitate interactions with hepatocytes, hepatic stellate cells, and endothelial cells. Under physiological conditions, these resident macrophages remove pathogens, cellular debris, and bacterial metabolites through phagocytosis (46). Experimental models show that KCs can be activated by different stress factors in MASH. Saturated free fatty acids and gut-derived LPS can activate them through the TLR4-MD2 complex, triggering ROS production and the secretion of proinflammatory cytokines such as IL-1β and TNF-α (24, 47). Moreover, DAMPs released from injured hepatocytes stimulate macrophages through pattern recognition receptors (PRRs) such as CD36 and TLR4 (48) (**Figure 2A**). Lipotoxic stress induces mitochondrial dysfunction and activates the NLRP3 inflammasome, promoting a proinflammatory phenotype (49) (**Figure 2B**).

During early disease stages, macrophages amplify hepatocyte injury and inflammation. As the disease progresses, subsets with specialized roles in lipid metabolism and extracellular matrix (ECM) remodeling emerge (50). Therefore, macrophages in MASH do not function as a homogeneous population, but multiple subsets are involved. This diversity is partly attributable to the sustained recruitment of circulating monocytes into the liver in response to cytokines and chemokines, such as CCL2, released by activated Kupffer cells and other cell populations (**Figure 2C**). Indeed, in patients with MASH, infiltration of CCR2^+^ monocyte-derived macrophages (MDMs) correlates with disease severity (41). In murine models of MASH, these recruited monocytes can partially replenish the resident Kupffer cell compartment, whose self-renewal capacity is compromised by hepatic lipid overload (51). In the human liver, MDMs can acquire specialized phenotypes, including lipid-associated macrophages (LAMs) and scar-associated macrophages (SAMs). The shared expression of markers such as triggering receptor expressed on myeloid cells 2 (TREM2) and CD9 makes it difficult to determine whether these populations represent truly distinct subsets, but their distinct localization within the liver suggests potential functional differences (44, 52).

LAMs are involved in the management of hepatic lipid overload. In MASH, dysregulated cholesterol metabolism leads to excessive esterification of free cholesterol within hepatocyte lipid droplet membranes. When the storage capacity is exceeded, accumulated cholesterol crystallizes, leading to hepatocyte apoptosis and the release of cholesterol-crystal-laden lipid droplets (53). LAMs accumulate around dying steatotic hepatocytes and expelled lipid droplets, forming crown-like structures (CLSs) (**Figure 2D**) (54, 55). Within these structures, macrophages secrete lysosomal enzymes, lipases, and lipid-binding proteins to promote lipid droplet degradation (56, 57). Indeed, experimental evidence in mice indicates that LAMs are key mediators of the hepatic histological recovery after bariatric surgery (58).

In contrast, SAMs accumulate in fibrotic regions and exhibit a pro-fibrogenic transcriptomic profile (52). The contribution of macrophages to hepatic fibrosis is complex. Following hepatic injury, hepatic stellate cells transition from quiescent pericytes to activated myofibroblast-like cells, producing collagen-rich extracellular matrix, proteoglycans, glycosaminoglycans, and matrix metalloproteinase inhibitors (59). Macrophages facilitate this activation by releasing pro-fibrogenic mediators, including TGF-β1, VEGF, and galectin-3 (41, 60). At later stages, SAMs may sustain the fibrogenic response (**Figure 2E**). Although fibrosis is a major risk factor for liver-related mortality, it initially serves as a wound-healing response to preserve tissue integrity and represents the common endpoint of most chronic inflammatory injuries (61). Murine studies indicate that fibrotic livers exhibit increased resistance to toxic insults compared to non-fibrotic livers, and this cytoprotective effect is lost upon macrophage depletion (62). This observation may explain why macrophages with anti-inflammatory properties can also promote fibrogenesis during chronic injury (63, 64). The life-threatening problem arises when persistent hepatic insults prevent the normal resolution of the wound healing process, leading to excessive scar tissue accumulation. Indeed, once hepatic insults cease, MDMs may contribute to fibrosis regression. In murine models of liver fibrosis, Ly-6C^lo^ macrophages promote collagen degradation by releasing matrix metalloproteinases (MMP) such as MMP9 and MMP12 (65) (**Figure 2F**). This activity has been linked to TREM2^+^ macrophages, which become the dominant subset during the remission phase of MASH in mice (54). Furthermore, in a mouse model of MASH, activated HSCs demonstrated a close association with macrophages within hepatic CLSs. HSCs would transfer extracellular vesicles to neighboring macrophages, promoting an anti-fibrotic phenotype characterized by the upregulation of the collagen protease cathepsin K (CTSK) (66).

TREM2 is a lipid-sensing receptor that regulates macrophage responses to extracellular lipid accumulation. Its expression in LAMs and SAMs highlights its relevance in the context of MASH. Beyond its possible role in shaping macrophage phenotype, both clinical and experimental evidence indicate that TREM2 may serve as a biomarker for MASH progression. Studies using mouse and cellular models indicate that prolonged hypernutrition increases hepatic concentrations of TNF-α and IL-1β, which promote TREM2 shedding via ADAM17-dependent proteolytic cleavage. The subsequent reduction in membrane-bound TREM2 impairs macrophage efferocytosis, sustains chronic inflammation, and contributes to the progression from simple steatosis to MASH (67). This cleavage releases soluble TREM2 (sTREM2) into the circulation. Elevated sTREM2 levels have been shown to distinguish individuals with MASH from those with simple steatosis or healthy controls (67–69). However, the potential of sTREM2 as a biomarker requires further validation in large patient cohorts.

The analysis of hepatic macrophages in MASH demonstrates that immune cell populations are heterogeneous and integrated by distinct subsets. These subsets have divergent roles, either pathogenic or reparative, in hepatic disease. Their precise characterization is therefore necessary both to understand the immunometabolic mechanisms that drive MASH and to determine how each subset could be therapeutically targeted. These considerations also apply to the remaining immune cell populations discussed in this review.

### 3.2 The unresolved role of dendritic cells

**Figure 3:**
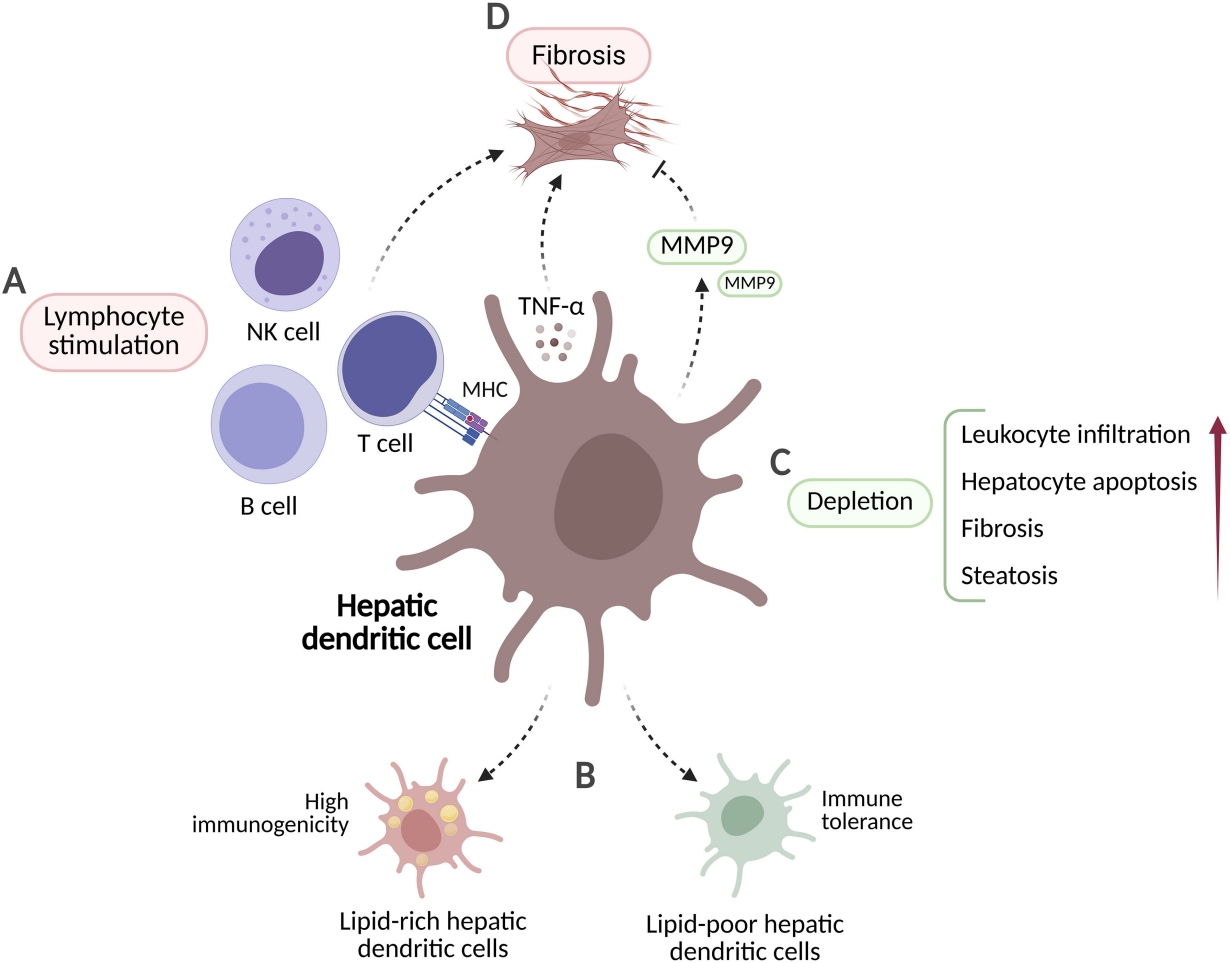
Potential functions of dendritic cells in MASH. **(A)** Dendritic cells facilitate inflammation by activating T cells, B cells, and natural killer (NK) cells through MHC-I and MHC-II interactions. **(B)** Lipid-rich hepatic dendritic cells exhibit elevated immunogenicity and proinflammatory activity, whereas lipid-poor hepatic dendritic cells contribute to tolerogenic responses. **(C)** Dendritic cell depletion promotes leukocyte infiltration, hepatocyte apoptosis, fibrosis, and steatosis. **(D)** TNF-α secretion by dendritic cells drives fibrogenesis, whereas matrix metalloproteinases (MMPs) contribute to fibrosis resolution. Created in BioRender. Joven, J. (2026) https://BioRender.com/5vfap6h.

Dendritic cells are specialized hematopoietic cells that originate in the bone marrow. The classification of these cells is based on their developmental lineage, surface markers, and function. Conventional dendritic cells (cDCs) migrate to lymphoid and non-lymphoid organs and mature into conventional type 1 (cDC1) and type 2 (cDC2) dendritic cells (70). Under conditions of hepatic homeostasis, dendritic cells exhibit a phenotype that supports immunologic tolerance, characterized by poor antigen endocytosis capacity, low T-lymphocyte stimulation activity, and induction of regulatory T cells (71). Upon exposure to danger signals, dendritic cells migrate to lymphoid organs and present antigens on MHC-I or MHC-II molecules to T and B lymphocytes as well as natural killer (NK) cells (72, 73) (**Figure 3A**). In the liver of both mice and humans, two different dendritic cell populations are distinguished by their lipid content. Lipid-rich hepatic dendritic cells exhibit high immunogenicity through TNF-α secretion. This subset efficiently activates T cells, NK cells, and NKT cells. In contrast, lipid-poor dendritic cells facilitate the induction of regulatory T cells and promote T cell anergy to tumor antigens (74) (**Figure 3B**).

The behavior of dendritic cells in MASH is poorly characterized. In patients with MASH, circulating cDC2 frequencies are elevated, while cDC1 frequencies show an inverse correlation with disease severity. Accordingly, in murine models of MASH, intrahepatic cDC2 frequencies increase while cDC1 frequencies decrease (75). Most mechanistic evidence on dendritic cell function in MASH is derived from experimental models and is still inconclusive. Some studies indicate a protective role. For example, depletion of dendritic cells in transgenic mice fed a methionine- and choline-deficient (MCD) diet led to increased hepatic leukocyte infiltration, enhanced hepatocyte apoptosis, and accelerated fibrogenesis (76). Similarly, in murine MASH models, loss of CD103^+^ cDC1 exacerbated steatosis and lobular inflammation, whereas adoptive transfer of these cells reduced hepatic CCL2 expression and monocyte recruitment (77) (**Figure 3C**). In contrast, XCR1^+^ cDC1 were associated with MASH progression in mice (78). The contribution of dendritic cells to hepatic fibrosis is also debated. Evidence from murine models of liver fibrosis suggests that dendritic cells promote hepatic stellate cell activation through TNF-α secretion and direct cell contact during chronic inflammation (79). Dendritic cells may also indirectly contribute to fibrogenesis by regulating profibrotic immune cell populations, such as CD8^+^ T cells (80). In contrast, a study using a carbon tetrachloride (CCl_4_)-induced murine model of fibrosis demonstrated that dendritic cell activation and MMP-9 secretion facilitate regression of fibrosis following cessation of liver injury (81) (**Figure 3D**).

The relevance of dendritic cells to human MASH is still unclear, and evidence from murine models is contradictory. Understanding the roles of dendritic cells in MASH requires precise characterization of their subsets in experimental models and rigorous validation of these findings in human MASH.

### 3.3 Neutrophils: at the crossroads of injury and repair

Neutrophils are primary effectors of innate immunity that mediate both acute and chronic inflammatory responses. These cells originate in the bone marrow, circulate systemically, and conduct immune surveillance. Under physiological conditions, neutrophils can exit the bloodstream to enter tissues, but their recruitment intensifies in response to infection or injury (82). Neutrophils have substantial immunoregulatory capacity. These cells interact with dendritic cells, macrophages, natural killer cells, and conventional lymphocytes to initiate, amplify, or inhibit immune responses, depending on the pathogenic context (83). However, the altered tissue microenvironment characteristic of chronic metabolic and inflammatory diseases significantly impairs neutrophil function, resulting in aberrant responses and tissue damage.

The contribution of neutrophils to MASH appears to depend on disease stage. In early stages, neutrophil depletion in murine models of diet-induced liver injury provides anti-inflammatory effects. In contrast, during disease remission, neutrophil depletion impairs hepatic recovery (40).

In the liver, neutrophils can release granules containing myeloperoxidase, lipocalin-2, metalloproteases, and neutrophil serine proteases such as proteinase 3 and neutrophil elastase. These granule proteins and proteolytic enzymes contribute to ROS generation, ECM remodeling, hepatocyte injury, insulin resistance, and the regulation of other immune cells’ activity. Neutrophils also secrete cytokines and chemokines that recruit monocytes, lymphocytes, and additional neutrophils to the liver, consequently amplifying the inflammatory response (84, 85). The Neutrophil-to-Lymphocyte ratio in blood has been identified as a non-invasive biomarker of systemic inflammation that is associated with the progression of MASH (86, 87). Neutrophil extracellular traps (NETs), composed of chromatin, granular proteins, and histones, are released during infections to trap and neutralize pathogens. However, in the context of chronic sterile hepatic inflammation, NETs contribute to cytokine production, monocyte recruitment, and fibrosis development (88). The exposure of primary mouse HSCs to isolated NETs enhanced mitochondrial respiration and aerobic glycolysis through the toll-like receptor 3/cyclooxygenase-2 pathway. This immunometabolic alteration promoted HSC activation and a fibrogenic response. These findings were further supported in a murine model fed a Western diet and treated with CCl_4_ (89). Additionally, NETs generation has been linked to the progression of MASH to HCC in mice (90). Indeed, the balance between NETs release and clearance, as well as the timing of these processes, is a major factor influencing disease progression (91).

**Figure 4:**
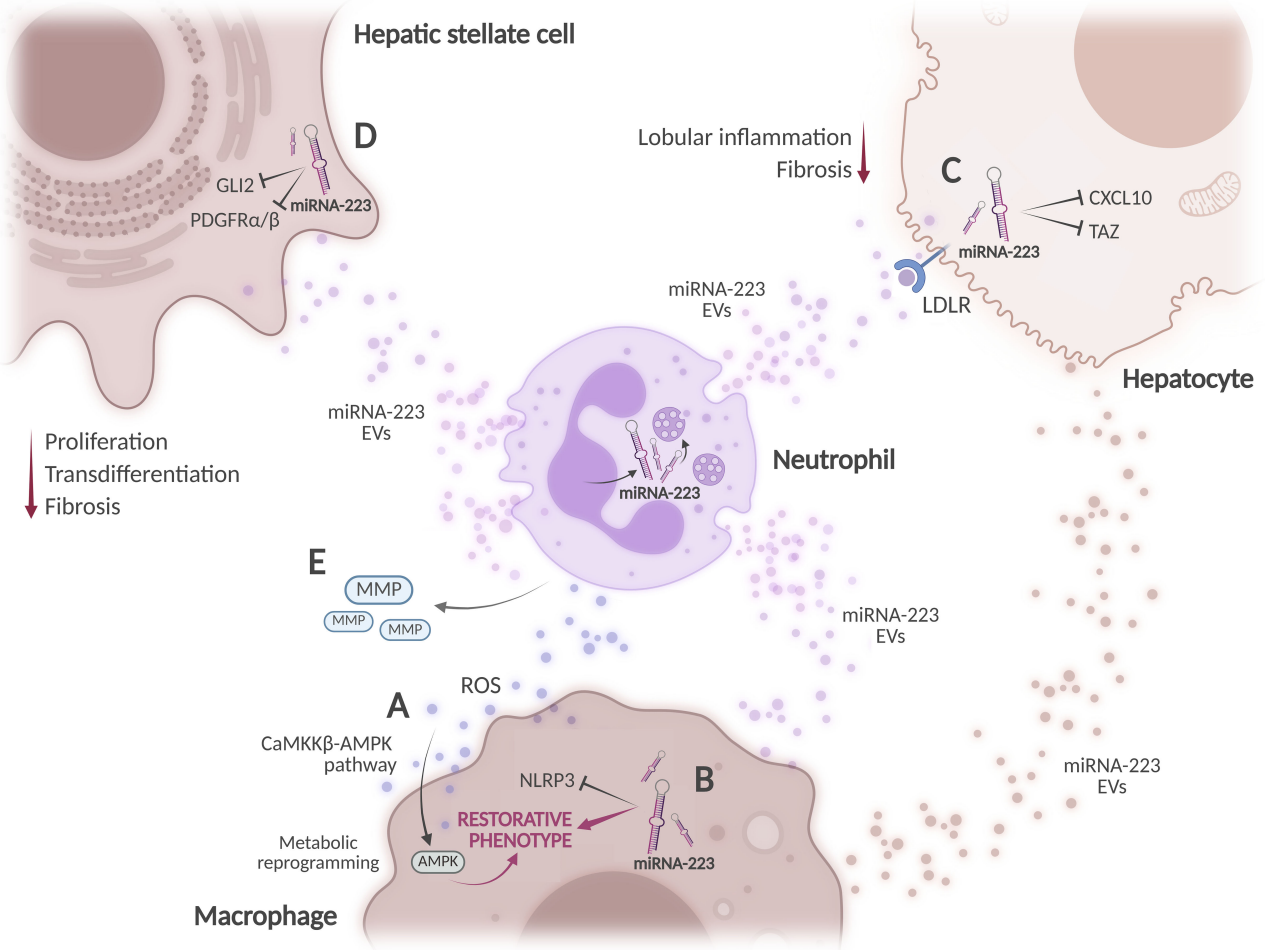
Reparative functions of neutrophils in MASH. **(A)** Neutrophil-derived reactive oxygen species (ROS) can induce metabolic reprogramming in macrophages via AMPK activation, driving a shift toward a restorative phenotype. **(B)** Neutrophils deliver miRNA-223 to hepatic macrophages through extracellular vesicles, facilitating anti-inflammatory phenotype polarization and inhibiting NLRP3 inflammasome activation. Macrophages also release miR-223-enriched extracellular vesicles that reach hepatocytes. **(C)** Hepatocytes internalize miRNA-223-containing vesicles via the LDL receptor (LDLR). miRNA-223 inhibits oncogenic and proinflammatory gene expression. **(D)** miRNA-223 attenuates hepatic stellate cell activation by modulating gene expression. **(E)** Neutrophil-derived matrix metalloproteinases (MMPs) may contribute to fibrosis remission. Created in BioRender. Joven, J. (2026) https://BioRender.com/e1t720x.

Despite their pathogenic roles, neutrophils can also contribute to tissue repair through different mechanisms (92, 93). Similar to macrophages, neutrophils form crown-like structures around steatotic hepatocytes in patients with morbid obesity. Their abundance correlates with body mass index (BMI) and markers of hepatocellular injury (94). Given the potential protective functions of macrophage crown-like structures, their role warrants further investigation. Evidence from a murine model of acetaminophen-induced acute liver injury, supported by *ex vivo* studies with macrophages, suggests that neutrophil-derived ROS can promote metabolic reprogramming in macrophages by activating AMPK via the Ca^2+^-CaMKKβ pathway. As a result, macrophages would shift toward restorative phenotypes that contribute to tissue regeneration following cessation of the hepatic insult (95) (**Figure 4A**). Central to neutrophil-mediated protection is miRNA-223, a neutrophil-derived microRNA that orchestrates anti-inflammatory responses (96). Neutrophils may transfer miRNA-223 to liver macrophages, hepatocytes, and HSCs through extracellular vesicles during both active disease progression and resolution. Preclinical evidence suggests that miRNA-223 promotes macrophage polarization towards a reparative phenotype while attenuating proinflammatory pathways, such as NLRP3 inflammasome activation (97) (**Figure 4B**). In hepatocytes, neutrophil-derived miRNA-223 inhibits oncogenic and inflammatory gene expression, especially targeting CXCL10 and TAZ, both implicated in inflammation and MASH progression (**Figure 4C**). *In vitro*, macrophages can also release miRNA-223-enriched extracellular vesicles that reach hepatocytes (98). Additionally, miRNA-223 exhibits anti-fibrotic properties by downregulating the expression of GLI2 and PDGF receptors (PDGFRα and PDGFRβ) in HSCs, reducing their activation (**Figure 4D**). TAZ inhibition in hepatocytes also reduces the activation of HSCs by decreasing Indian hedgehog (IHH) signaling (99). Experimental evidence from murine CCl_4_-induced liver fibrosis models suggests that neutrophils may also participate in fibrosis resolution by secreting MMP8 and MMP9 (100) (**Figure 4E**).

Neutrophils should not be viewed merely as amplifiers of liver injury. However, it remains uncertain whether the transition from damaging to reparative activities results from the functional reprogramming of neutrophils or the emergence of distinct subsets at different stages of the disease. This distinction would determine whether neutrophil-targeted therapies should focus on modulating cell function or selectively targeting specific populations. Indeed, although not in the context of MASH, distinct neutrophil populations have been identified that differ in phenotype, maturation status, and function (101). At first glance, therapeutic strategies targeting neutrophils in MASH could involve limiting NET formation and promoting the protective functions of miRNA-223. However, evidence supporting reparative roles for neutrophils is largely derived from preclinical models, and its relevance to human MASH remains to be established. In addition, the impact of metabolic dysfunction on neutrophils is very poorly characterized. A better understanding of their immunometabolic reprogramming may help clarify their heterogeneous activity in MASH.

### 3.4 Innate lymphoid cells

Innate lymphoid cells are a family of tissue-resident immune effectors that lack the genetically recombined antigen receptors found in T and B lymphocytes. This heterogeneous group includes five principal subsets: natural killer (NK) cells, type 1, 2, and 3 innate lymphoid cells (ILC1, ILC2, ILC3), and lymphoid tissue inducer (LTi) cells. ILC1s, ILC2s, and ILC3s mirror the functions of T helper 1 (Th1), Th2, and Th17 CD4^+^ cells, respectively, whereas NK cells exhibit cytotoxic functions analogous to those of CD8^+^ T cells. Unlike NK cells, which can circulate, ILCs predominantly maintain tissue residency and establish specialized niches within organs such as the gut, skin, lung, liver, and adipose tissue (102, 103).

NK cells comprise approximately 30-40% of intrahepatic lymphocytes (104, 105). In response to cytokine and microenvironmental cues, NK cells can undergo metabolic reprogramming. They may increase their reliance on oxidative phosphorylation to support IFN-γ production and cytotoxicity, or enhance glucose uptake and glycolysis to meet higher energetic demands. mTOR is a central regulator of this adaptation (106, 107). However, in obesity, this metabolic flexibility is impaired. Experimental evidence from murine and cellular models indicates that lipid accumulation in NK cells, driven by PPAR-dependent pathways, suppresses mTOR signaling. This defect is associated with reduced cytotoxicity and impaired anti-tumor activity (108). While these findings are not established in MASH models, they provide a mechanistic basis for hypothesizing that these immunometabolic alterations are relevant to obesity-associated liver disease.

**Figure 5:**
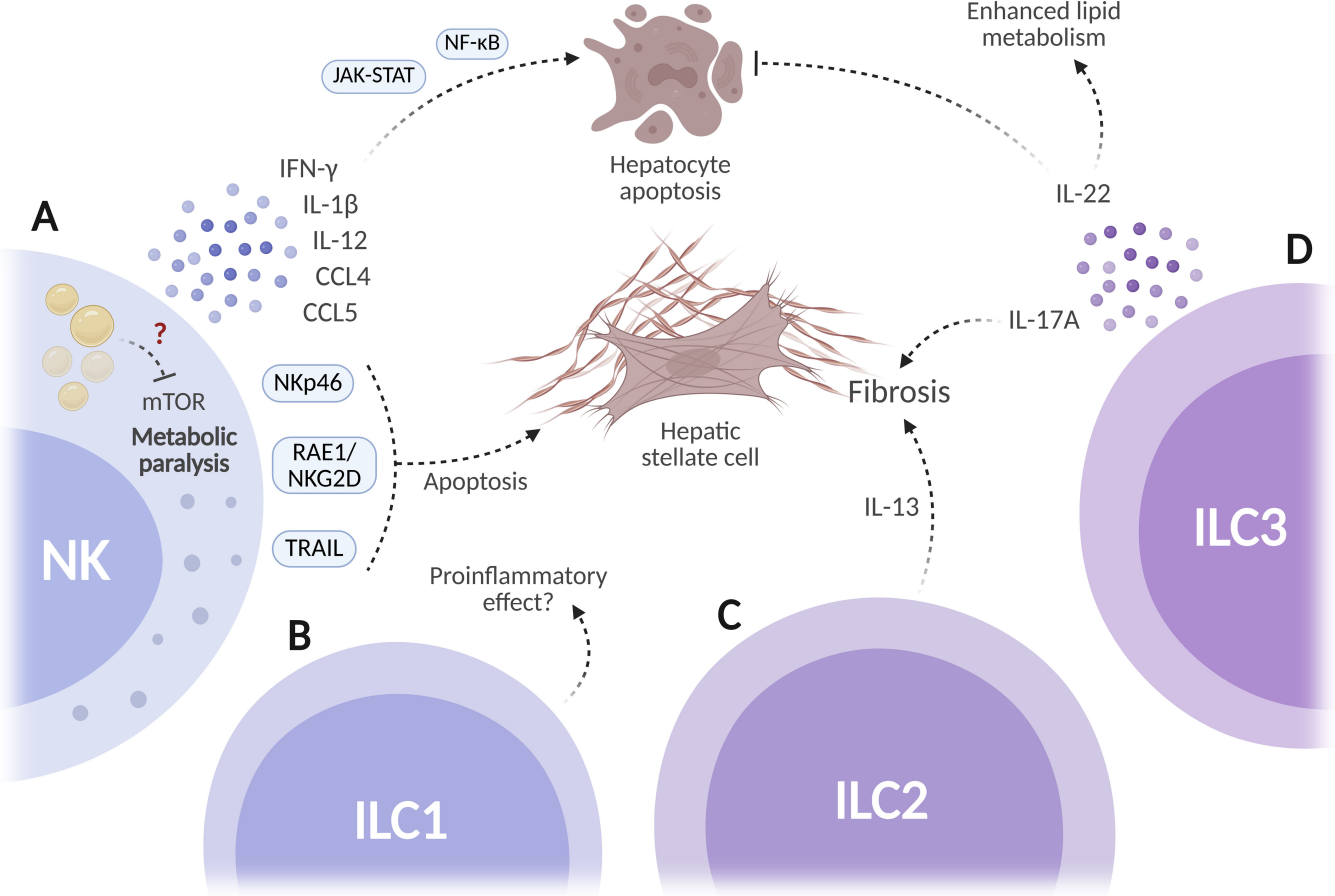
Potential functions of innate lymphoid cells (ILCs) in MASH. **(A)** Natural killer (NK) cells may undergo metabolic paralysis due to lipid accumulation. These cells can promote hepatocyte death but also induce hepatic stellate cell apoptosis through different interactions. **(B)** ILC1s may exert proinflammatory effects in liver disease. **(C, D)** ILC2 and ILC3 subsets may contribute to fibrosis progression, but ILC3-derived IL-22 could enhance lipid metabolism and inhibit hepatocyte apoptosis. Created in BioRender. Joven, J. (2026) https://BioRender.com/yf8igaj.

The role of NK cells in MASH is poorly described. In murine MASH models, hepatic NK cells increase in number and acquire an activated phenotype characterized by higher expression of NKG2D and CD107a. These cells secrete proinflammatory cytokines, including IFN-γ, IL-1β, IL-12, CCL4, CCL5, and GM-CSF, which are associated with hepatocyte damage, increased ROS production, apoptosis, and activation of JAK-STAT1/3 and NF-κB signaling pathways (109). With respect to fibrosis, *in vitro* evidence suggests that NK cells may exert anti-fibrotic effects by eliminating activated HSCs through NKp46/NCR1-dependent, RAE1/NKG2D-dependent, and TRAIL-dependent mechanisms (110, 111) (**Figure 5A**).

Current evidence regarding other ILC subsets in MASH is very limited and primarily based on experimental models. To date, no studies have specifically addressed the role of ILC1s in MASH. These cells may help sustain the hepatic proinflammatory state, but their partial phenotypic and functional overlap with NK cells makes it challenging to attribute specific effects to ILC1s (112) (**Figure 5B**). Evidence from murine models of hepatic fibrosis suggests that ILC2s may contribute to fibrogenesis through IL-13-dependent mechanisms (113) (**Figure 5C**). ILC3s may have a protective role, since mice deficient in ILC3s develop more severe hepatic steatosis and fibrosis than controls. Additionally, IL-22 produced by ILC3s confers hepatoprotective effects by regulating lipid metabolism and preventing hepatocyte apoptosis (114). However, ILC3s accumulate in fibrotic human livers and may facilitate fibrogenesis by secreting IL-13 (115) (**Figure 5D**).

The contribution of ILCs to MASH development is still far from clear. A better understanding of NK cell metabolic reprogramming in the diseased liver may reveal novel immunometabolic therapeutic opportunities for MASH, and a deeper characterization of their potential anti-fibrotic activity could provide new strategies to target hepatic fibrosis.

## 4 Adaptive immunity in MASH

The pathogenesis of MASH comprises complex interactions between innate and adaptive immune components that sustain hepatic inflammation. Adaptive immune responses can either worsen tissue damage, such as through cytotoxic T cell and B cell activity, or facilitate repair processes (37).

### 4.1 T cell-mediated immune responses

T cells function as key regulators of adaptive immune responses. Through the T-cell receptor (TCR) and CD3 co-receptor complex, T cells recognize antigens presented on MHC class I or II molecules by antigen-presenting cells and initiate downstream effector functions (116). Distinct T cell subsets contribute variably to the development of MASH.

#### 4.1.1 CD4^+^ T cells: progressive transition toward profibrogenic responses

CD4^+^ T helper cells use CD4 as a co-receptor with the TCR to enhance antigen recognition specificity. Upon activation, naive CD4^+^ T cells differentiate into distinct T helper (Th) subsets or regulatory T cells, a process influenced by the local cytokine environment (117).

**Figure 6:**
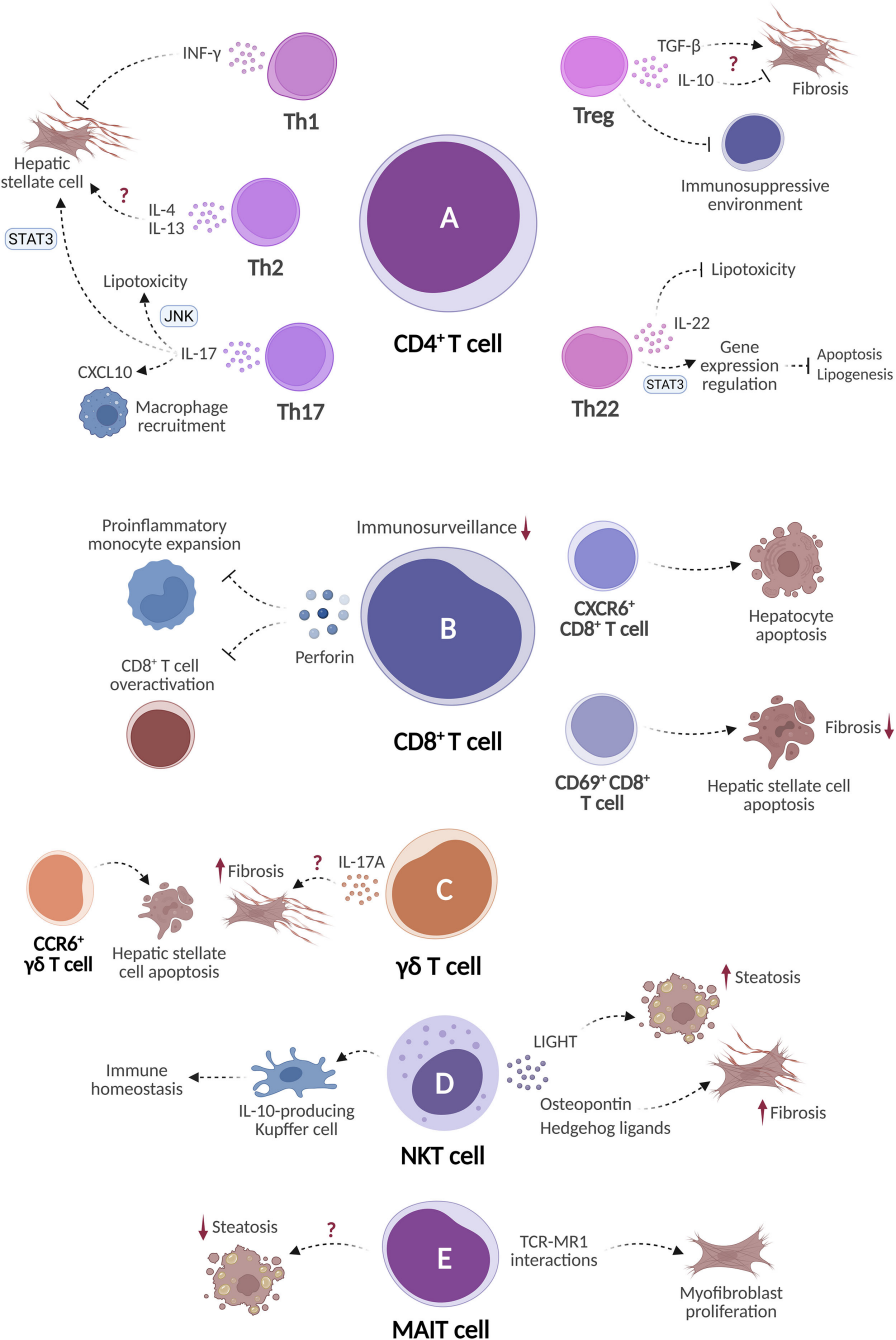
Heterogeneity and functional complexity of T lymphocyte subsets in MASH progression and remission. T lymphocytes are classified as either conventional or unconventional. Conventional T cells include **(A)** CD4^+^ helper T cells, which differentiate into distinct functional subsets (Th1, Th2, Th17, Th22, and Treg), and **(B)** CD8^+^ cytotoxic T cells. Unconventional T cells, which constitute the majority of intrahepatic lymphocytes, include **(C)** γδ T cells, **(D)** natural killer T (NKT) cells, and **(E)** mucosal-associated invariant T (MAIT) cells. These subsets exhibit context-dependent roles in hepatic inflammation and fibrosis. Created in BioRender. Joven, J. (2026) https://BioRender.com/x4gaegy.

In advanced stages of MASH, the CD4^+^ T cell response becomes progressively dominated by proinflammatory and profibrogenic subsets. Th17 cells, characterized by IL-17 secretion, are the CD4^+^ T cell subset most consistently associated with profibrotic responses. Both preclinical and clinical studies show that progression from steatotic liver to MASH correlates with increased intrahepatic frequencies of IL-17^+^ CD4^+^ T cells (42, 118). Mechanistic studies in mice indicate that IL-17 contributes to hepatic inflammation by inducing CXCL10-dependent macrophage accumulation and enhancing collagen secretion by HSCs via STAT3-dependent pathways (119–122). Furthermore, experiments using cultured mouse hepatocytes indicate that IL-17 can intensify palmitate-induced lipotoxicity by activating JNK signaling (123) (**Figure 6A**).

Th1 cells may also contribute to the development of this advanced inflammatory state, although the available evidence is less consistent than for Th17 cells. The frequency of peripheral blood Th1 cells is elevated in patients with MASH compared to healthy controls (124). Activated Th1 cells secrete proinflammatory cytokines, such as IFN-γ, IL-2, and TNF-α (116). IFN-γ may be an important mediator of MASH progression. In a murine steatohepatitis model induced by a methionine- and choline-deficient high-fat diet, IFN-γ deficiency attenuated hepatic inflammation, reduced hepatic stellate cell activation, and decreased liver fibrosis (125). Although IFN-γ is not produced exclusively by Th1 cells, its secretion by this subset may account for their detrimental role in MASH (**Figure 6A**).

The roles of Th2 and Th22 cells in MASH are less clearly defined. Patients with MASH exhibit increased Th2 cell frequency in peripheral blood (126). Although Th2-associated cytokines, such as IL-4 and IL-13, may promote fibrogenic responses, the overall contribution of Th2 cells to MASH is still inconclusive (127, 128) (**Figure 6A**). Th22 cells are characterized by their production of IL-22, a cytokine that has shown hepatoprotective effects in experimental models of liver disease. In mice with T cell-mediated hepatitis, IL-22 protected hepatocytes from injury by activating STAT3 signaling, which promoted cell survival through anti-apoptotic and mitogenic programs (129). In mouse hepatocyte cultures, IL-22 reduced palmitate-induced lipotoxicity via PI3K-mediated inhibition of JNK (123). Additionally, administration of recombinant IL-22 to high-fat diet (HFD)-fed mice decreased hepatic fat accumulation and downregulated the expression of lipogenic genes, including PPARα, PPARγ, and SREBP-1c (130) (**Figure 6A**).

The hepatic T helper cell compartment exhibits considerable heterogeneity. Therapeutic inhibition of the potential proinflammatory and profibrogenic activity of Th17 cells may be especially pertinent in advanced stages of MASH. For other T helper cell subsets, the available evidence is still too limited to support the development of targeted interventions. However, better characterization of IL-22 effects in MASH, as well as determination of the disease stage at which Th22 responses are most significant, may help clarify whether enhancing the activity of this subset is a viable therapeutic strategy.

Regulatory T (Treg) cells are a specialized subset of CD4^+^ T cells defined by the expression of the transcription factor forkhead box P3 (Foxp3) (131). These cells maintain immune tolerance to self-antigens and limit excessive activation of effector T cells, preventing tissue damage. In MASH, the behavior and function of Treg populations are unclear. Studies in murine models have reported both increased and decreased frequencies of circulating and hepatic Treg cells, indicating that their role may depend on the experimental setting or disease stage (132–134). The relative balance between Treg cells and effector T helper cell subsets appears to be more relevant than the absolute number of Treg cells. Patients with MASH exhibit increased Th2/Treg and Th17/Treg ratios in peripheral blood compared to healthy controls, and the hepatic Th17/Treg ratio is also elevated (42). Treg cells have been shown to protect against immune-mediated liver injury in experimental models of viral and autoimmune hepatitis (135, 136). Conversely, in diet-induced MASH mice lacking adaptive immunity, adoptive transfer of Treg cells worsened hepatic steatosis and increased serum ALT levels, suggesting a potentially detrimental role in this setting (137). The contribution of Treg cells to MASH-associated fibrosis is undefined. These cells may promote fibrosis through TGF-β secretion but could also facilitate its resolution via IL-10 (138). Additionally, evidence from murine models suggests that the accumulation of Treg cells in the liver may promote the progression from MASH to HCC by creating an immunosuppressive microenvironment (132) (**Figure 6A**).

Although Treg cells are traditionally recognized for their role in preventing excessive immune-mediated tissue damage, current evidence suggests that their effects in MASH are not uniformly beneficial. However, mechanistic data are very limited, and definitive conclusions regarding their contribution to MASH cannot yet be established.

#### 4.1.2 CD8^+^ T cells: maladaptive cytotoxicity in a metabolically altered liver

Current evidence points to a predominantly pathogenic role for CD8^+^ T cells in MASH. Hepatic infiltration of these cells increases in both murine models of MASH and patients with MASH-related cirrhosis (139, 140). Distinct CD8^+^ T cell subsets emerge during disease progression and remission. In both mice and humans, the frequency of dysfunctional CD8^+^ PD1^+^ T cells with impaired antitumor immunosurveillance capacity rises as MASH progresses (141). A complementary study demonstrated that the tissue-resident CXCR6^+^ CD8^+^ cell subset acquires auto-aggressive properties in MASH. IL-15-mediated downregulation of FOXO1 and upregulation of CXCR6 increases the sensitivity of these cells to metabolic cues such as acetate and extracellular ATP, promoting MHC class I-independent killing of hepatocytes (142). Consistent with the contribution of CD8^+^ T cells to hepatic injury, their depletion in obese mice reduces hepatic inflammation, HSC activation, and macrophage infiltration (140). However, not all CD8^+^ T cell functions are deleterious. In murine models of diet-induced MASH, loss of CD8^+^ T cell-derived perforin exacerbates liver injury and promotes the expansion of proinflammatory monocytes and overactivation of hepatic CD8^+^ T cells (143). Therefore, despite being a cytotoxic effector molecule, perforin may limit hepatic inflammation in MASH. Furthermore, during MASH remission in experimental models, tissue-resident memory CD69^+^ CD8^+^ T cells exert anti-fibrotic effects by interacting with HSCs and inducing their apoptosis (144) (**Figure 6B**).

The function of CD8^+^ T cells in MASH is conditioned by the hepatic microenvironment. Within the metabolically altered and inflamed liver, CXCR6^+^ CD8^+^ T cells are pathologically reprogrammed. A similar process may also occur in other subsets. These observations have important therapeutic implications, as they support selective targeting of pathogenic immunometabolic pathways that drive CD8^+^ T cell reprogramming, while preserving mechanisms involved in inflammation control and fibrosis resolution.

### 4.2 Unconventional T cells as potential regulators of hepatic fibrosis

Unconventional T cells constitute a functionally distinct group with a unique capacity to recognize non-peptidic antigens. γδ T cells respond rapidly to stress signals and infections. Natural killer T (NKT) cells modulate immune responses through the recognition of lipid antigens. Mucosal-associated invariant T (MAIT) cells are specialized in detecting microbial metabolites. Collectively, these subsets comprise the majority of intrahepatic T lymphocytes (145).

γδ T cells express a T cell receptor composed of γ and δ subunits. These cells recognize ligands independently of MHC molecules and secrete cytokines such as IFN-γ, IL-22, TNF-α, and IL-17A (145). In murine models of steatohepatitis, hepatic γδ T cell frequency increases due to both proliferation of resident populations and migration from lymphoid organs (146). These cells accumulate within fibrotic regions in the human and murine liver (147). In mice, cholesterol-induced stress ligands trigger NKG2D-mediated activation of γδ T cells and IL-17A secretion, which is a key mediator of fibrosis progression (38). In contrast, a study in murine models of CCl_4_-induced and MCD diet-induced hepatic injury identified a distinct CCR6^+^ γδ T cell subset expressing IL-17 and IL-22, which may limit advanced liver fibrosis by inducing HSC apoptosis through a cell-contact-dependent, FasL-mediated mechanism (148) (**Figure 6C**). Therefore, although γδ T cells appear to participate in hepatic fibrosis, their specific role is still unresolved.

NKT cells are a diverse group of innate-like T cells. Type 1 or invariant NKT (iNKT) cells express a semi-invariant αβ TCR that recognizes glycolipid antigens presented by the MHC class I-like molecule CD1d. Type 2 NKT cells or diverse NKT (dNKT) are also CD1d-restricted but display more diverse αβ TCR repertoires and distinct lipid antigen recognition properties. In humans, type 1 NKT cells constitute less than 1% of T cells, whereas type 2 NKT cells are more abundant (149). NKT cell frequencies increase with the severity of hepatic steatosis in both the liver and peripheral blood of patients (150, 151). In the early stages of MASH, NKT cells may exert protective functions. In a cellular co-culture system, primary hepatic iNKT cells enhanced IL-10 expression in the immunoregulatory KC-1 Kupffer cell subset through CD206-dependent crosstalk, contributing this way to immune homeostasis (152). Consistently, HFD-fed mice lacking NKT cells exhibited increased weight gain, hepatic steatosis and inflammation, and accelerated fibrogenesis (153, 154). However, as the disease progresses, NKT cells may transition toward pathogenic roles. In a long-term choline-deficient high-fat diet-fed murine model, NKT cell-derived LIGHT promoted fatty acid uptake in hepatocytes and contributed to steatosis (155). Furthermore, in mice fed an MCD diet, NKT cell accumulation in the liver correlated with increased fibrosis. These cells may promote HSC activation and fibrogenesis by secreting osteopontin and Hedgehog ligands (156, 157) (**Figure 6D**).

MAIT cells recognize microbial-derived vitamin B metabolites presented by MHC class I-related protein 1 (MR1) (145). Evidence for MAIT cell activity in MASH is limited and inconclusive. In patients with MASH, MAIT cells are reduced in peripheral blood but increased in the liver, where their abundance correlates with histological injury severity (158). In HFD-fed mice with a genetically determined increase in MAIT cells, reduced hepatic lipid accumulation and lower expression of lipogenic genes suggested a protective role for these cells during the early stages of disease (159). Conversely, in advanced stages of hepatic fibrosis, MAIT cells may exert profibrogenic effects. In patients with cirrhosis of alcoholic or MASH etiology, intrahepatic MAIT cells are reduced in abundance and are found mainly within fibrotic septa, in close proximity to myofibroblasts. In this setting, they acquire an exhausted phenotype but retain proinflammatory activity. Through TCR/MR1-dependent interactions, activated MAIT cells may contribute to fibrogenesis by promoting myofibroblast proliferation (**Figure 6E**) (160). In line with this potential profibrogenic role, MR1 blockade reduced MAIT cell activation and promoted histological regression of MASH-induced fibrosis in mice fed a choline-deficient L-amino acid-defined high-fat diet (161).

Current evidence indicates that γδ T cells and MAIT cells may be primarily involved in shaping the hepatic fibrotic response. Consequently, these cell populations may serve as potential therapeutic targets to limit fibrosis progression. In contrast, the specific roles of NKT cells are uncertain. It is unclear whether the diverse functions attributed to NKT cells reflect true functional heterogeneity or result from the coexistence of distinct NKT cell subsets. Since most available data originate from preclinical models, findings may be influenced by experimental conditions and require validation in human MASH.

### 4.3 B cells in disease progression and inter-organ communication

**Figure 7:**
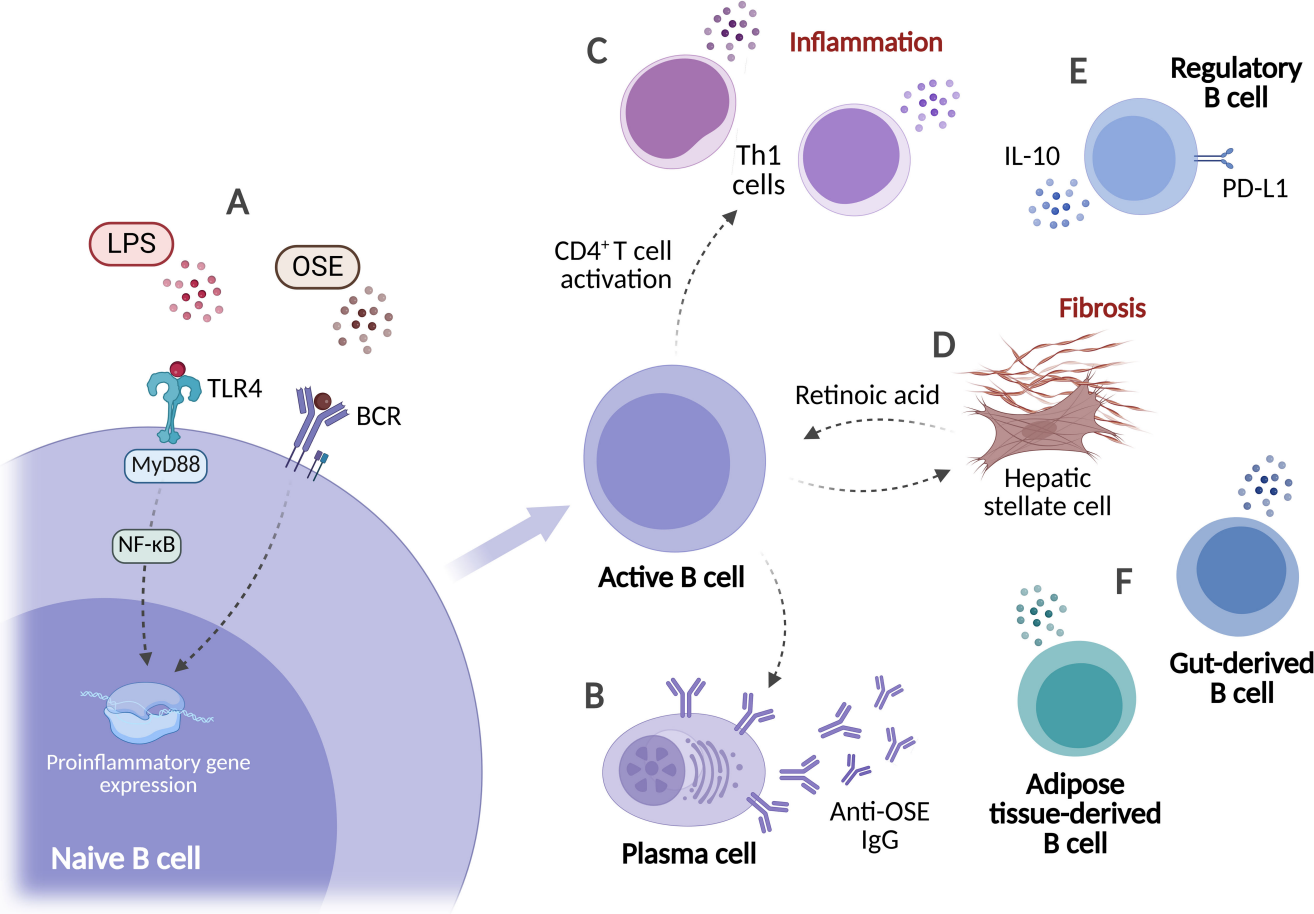
B cell activation and functional diversity in MASH. **(A)** Stress and damage signals activate B cells and induce proinflammatory gene expression. **(B)** Plasma cells generate IgG against oxidation-specific epitopes (anti-OSE), which are elevated in the circulation of patients with MASH. **(C)** B cells amplify hepatic inflammation by facilitating CD4^+^ T-cell activation and Th1 polarization. **(D)** B cells engage in reciprocal interactions with hepatic stellate cells, which promote B-cell activation and survival via retinoic acid secretion. **(E)** Regulatory B-cell subsets expressing PD-L1 and IL-10 may facilitate disease progression through immunosuppressive functions. **(F)** Extrahepatic B-cell populations from adipose tissue and gut-associated lymphoid tissues migrate to the liver and exacerbate hepatic inflammation. Created in BioRender. Joven, J. (2026) https://BioRender.com/pze6a3i.

The activation of B cells occurs during the early stages of steatohepatitis. In wild-type mice fed a MCD diet, intrahepatic B cells rapidly responded to hepatic injury and differentiated into plasma cells (162). These cells can be stimulated by both intrahepatic and extrahepatic signals. In mice with MASH, gut-derived microbial antigens promoted intrahepatic B cell activation through the combined action of TLR4/MyD88 signaling and B cell receptor-mediated antigen recognition (163). Oxidative stress within the liver generates oxidation-specific epitopes (OSE) that may further promote B-cell responses (**Figure 7A**). Patients with MASH exhibit increased circulating anti-OSE IgG, suggesting that these antigens may drive a humoral immune response (164) (**Figure 7B**). Higher circulating anti-OSE IgG levels are associated with a greater prevalence of T-cell-rich intrahepatic aggregates containing B cells in human liver biopsies (162).

Beyond antibody production, B cells may contribute to the inflammatory process of MASH by modulating T-cell responses. Indeed, B cell activation may precede the hepatic recruitment and activation of CD4^+^ and CD8^+^ T cells (162). In murine models of MASH, intrahepatic B cells have been shown to promote CD4^+^ T cell activation and differentiation toward a Th1 phenotype (163, 165) (**Figure 7C**). Accordingly, in mice fed a MCD diet, B cell deficiency was associated with reduced circulating anti-OSE IgG levels and decreased hepatic recruitment of CD4^+^ and CD8^+^ T cells (162).

B cells may also contribute to the progression of hepatic fibrosis. In a CCl_4_-induced mouse model of liver fibrosis, B-cell deficiency attenuated fibrotic progression. Indeed, B cells were proposed to promote fibrosis primarily through their ability to amplify hepatic inflammation, with MyD88 signaling required for fibrosis progression. This profibrotic effect may be reinforced by interactions with hepatic stellate cells, as *in vitro* co-culture experiments indicate that activated stellate cells support B-cell survival and activation through retinoic acid secretion (166) (**Figure 7D**). Similarly, in a long-term choline-deficient amino acid-defined diet murine model, which reproduces MASH-associated fibrosis, B cell deficiency was also associated with reduced collagen deposition over time (162). Clinical observations are consistent with these findings. Serum levels of B-cell-activating factor (BAFF), a cytokine involved in B-cell maturation, are elevated in patients with MASH and correlate with advanced fibrosis (167). Furthermore, BAFF deficiency or pharmacological BAFF inhibition in murine models of metabolic steatohepatitis reduces adipose tissue and hepatic inflammation and attenuates hepatic steatosis (162, 168).

As with previously described immune cell types, different B cell subsets may participate in MASH. Besides conventional B cells, two B regulatory cell (Breg) populations were identified in the liver of HFD-fed murine models. Both subsets displayed an immunosuppressive phenotype characterized by increased PD-L1 and IL-10 expression, which may contribute to the progression of liver disease (169) (**Figure 7E**). Furthermore, B-cell subsets from extrahepatic organs may be found in the liver during MASH progression. In mice fed an HFD, B lymphocytes from mesenteric adipose tissue showed a tendency to migrate toward the liver and were proposed to contribute to the hepatic inflammatory response (170). Similarly, in mice, gut-derived B cells activated within intestinal lymphoid tissues migrate to the liver during disease progression (**Figure 7F**). *Ex vivo*, these intestinal B cells promoted CD8^+^ T-cell hyperactivation via an antigen-independent mechanism that required direct cell-cell interactions (171). Therefore, B cells may be central orchestrators of an inter-organ inflammatory network that links the gut, adipose tissue, and liver.

B cells connect multiple pathogenic layers of the disease. Rather than being terminal effectors of liver injury, these cells may function as early amplifiers of the inflammatory response that sustains disease progression. The capacity of extrahepatic B-cell populations to migrate to the liver supports the systemic nature of the inflammatory process in MASH. Although modulating B-cell-driven inflammatory amplification may hold therapeutic value, current evidence is still insufficient to support B cells as a direct therapeutic target, especially given that most mechanistic data derive from murine models and their contribution to human disease remains uncertain.

## 5 Combining metabolic and immune interventions for MASH

Current treatment strategies for MASH remain centered on weight-loss interventions and metabolic correction, overlooking immune dysregulation as an important factor in disease progression. Lifestyle modification remains the first-line approach for weight reduction, while bariatric surgery is considered the most effective intervention for severe obesity (172, 173). Pharmacological agents that promote weight loss have also shown efficacy in managing MASH. In 2025, semaglutide, a GLP-1 receptor agonist, was approved for MASH as Wegovy (174). Although these interventions may have secondary effects on the hepatic immune environment, their primary therapeutic mechanisms do not directly address immune dysregulation. The immunometabolic nature of MASH requires therapeutic strategies that target simultaneously metabolic and immunological pathways. Such strategies have not yet been established, but some approaches move in this direction (175).

Targeting nuclear receptors that regulate cellular metabolism has emerged as a promising therapeutic strategy for MASH. Resmetirom, a liver-directed thyroid hormone receptor-β (THR-β) agonist, became the first pharmacological therapy approved for MASH after a phase III trial demonstrated increased rates of disease resolution and fibrosis improvement (176). In addition to THR-β, other nuclear metabolic receptors, such as peroxisome proliferator-activated receptors (PPARs) and the farnesoid X receptor (FXR), have been identified as possible therapeutic targets. However, these agents were not designed to directly target immune cells. Consequently, the effects of these metabolic therapies on hepatic immune cell populations still need to be examined. The development of specific agonists that reprogram immune-cell metabolism in the liver to limit their pathogenic functions represents a potential therapeutic strategy for MASH.

A related line of evidence involves sodium-glucose cotransporter 2 inhibitors (SGLT-2i). Although these agents are not considered immune-targeted therapies, recent studies have demonstrated their immunometabolic effects in preclinical models of MASH. For example, empagliflozin suppresses the activation and infiltration of auto-aggressive CXCR6^+^ CD8^+^ T cells through ketogenesis-dependent mechanisms (177). Dapagliflozin and canagliflozin, two additional SGLT-2i, inhibit glycolysis in proinflammatory macrophages and promote a shift toward oxidative metabolism, consistent with polarization toward an anti-inflammatory phenotype (178).

Immune-targeted therapies have been developed in parallel to metabolic approaches. Cenicriviroc, a dual CCR2/CCR5 antagonist, was designed to inhibit monocyte recruitment to the liver via the CCL2-CCR2 axis. However, it was discontinued as monotherapy for MASH after phase III trials failed to demonstrate sustained antifibrotic efficacy (179). Broad suppression of monocyte infiltration may have unintended consequences, such as inhibiting the activity of reparative cell subsets. Therefore, strategies that modulate immune infiltration and function must balance the suppression of pathological inflammation with the preservation of protective immune mechanisms.

An alternative line of investigation has focused on using immune cells as direct antifibrotic mediators. In preclinical models, chimeric antigen receptor (CAR)-macrophages (CAR-M) targeting the urokinase plasminogen activator receptor (uPAR), a cell-surface receptor upregulated in HSCs in cirrhotic human livers, demonstrated efficacy in reducing liver fibrosis (180). A recent study engineered CAR-T cells targeting fibroblast activation protein (FAP), a cell-surface protein overexpressed on activated HSCs, and reported antifibrotic effects across four murine models of liver fibrosis, including MASH-associated fibrosis (181). However, the clinical effectiveness of these approaches remains to be determined.

The interactions between metabolic and immune factors are complex and not yet fully understood. Therefore, the development of combination therapies requires a comprehensive understanding of immune functions in metabolic hepatic disease, as extensively discussed in our recent review (175).

## 6 Framing immune complexity in MASH

MASH is a complex disorder that goes beyond simple metabolic explanations. Immune responses in the liver transition from tolerance to activation, which may result in tissue repair and resolution or, if persistent, lead to fibrosis and liver dysfunction. The hepatic microenvironment in MASH induces metabolic changes in immune cells that determine their functions. Conversely, dysfunctional immune activity perpetuates hepatic metabolic issues. Metabolic and immune dysregulation reinforce each other. Therefore, MASH should be recognized as an immunometabolic disease, not a combination of metabolic and inflammatory alterations.

Immune responses in MASH may contribute not only to disease progression but also to remission. Understanding the complex activity of immune cells in MASH requires moving beyond assigning fixed functions to broad immune cell categories and recognizing that each one consists of multiple subsets with distinct, context-dependent functions. It is also essential to elucidate how the hepatic microenvironment influences the functional state of immune cells through metabolic reprogramming. Currently, most evidence on this influence comes from experimental models, so the relevance of these immunometabolic mechanisms to human disease remains to be established.

Therapeutic strategies for MASH should target both metabolic and immunological pathways. Effective approaches must also consider the complicated interactions among immune cell subsets and whether each one exerts protective or detrimental effects. However, focusing therapeutic efforts solely on the liver would overlook the systemic nature of MASH. Disruption of the gut barrier, adipose tissue inflammation, and hepatic immune activity are interconnected and collectively drive disease progression. Therefore, proinflammatory stimuli originating outside the liver should be considered in the development of effective therapies.

Recognizing the immunometabolic nature of MASH is necessary to guide future clinical research. An in-depth understanding of the molecular connections between metabolic and immunological processes is fundamental for improving the management of this complex disease.

## Glossary

AC: adenylyl cyclase
ACC1: acetyl-CoA carboxylase 1
ACLY: ATP-citrate lyase
ADAM17: a disintegrin and metalloprotease 17
ALT: alanine aminotransferase
AMPK: AMP-activated protein kinase
APCs: antigen-presenting cells
AST: aspartate aminotransferase
ATGL: Adipose triglyceride lipase
ATP: adenosine triphosphate
BAFF: B cell-activating factor
BCR: B cell receptor
BMI: body mass index
Breg: regulatory B cell
β-AR: beta-adrenergic receptor
CaMKKβ: calcium/calmodulin-dependent protein kinase kinase β
CAR: chimeric antigen receptor
CCL: C-C motif chemokine ligand
CCL2: C-C motif chemokine ligand 2
CCl4: carbon tetrachloride
CCR2: C-C motif chemokine receptor 2
CD: cluster of differentiation
CD1d: non-classical MHC class I-like molecule
cDC: conventional dendritic cell
cDC1: conventional type-1 dendritic cell
cDC2: conventional type-2 dendritic cell
CLS: crown-like structure
CXCL: C-X-C motif chemokine ligand
CXCR: C-X-C motif chemokine receptor
DAG: diacylglycerol
DAMPs: damage-associated molecular patterns
DC: dendritic cell
DGAT: diacylglycerol acyltransferase
DNA: deoxyribonucleic acid
dNKT: diverse natural killer T cell
DNL: *de novo* lipogenesis
ECM: extracellular matrix
ER: endoplasmic reticulum
EV: extracellular vesicles
FAP: fibroblast activation protein
FASN: Fatty acid synthase
FFA: free fatty acid
FOXO1: forkhead box protein O1
Foxp3: forkhead box protein 3
FXR: farnesoid X receptor
G3P: glycerol-3-phosphate
GCK: glucokinase
GLI2: GLI Family Zinc Finger 2
GLP-1: glucagon-like peptide-1
GLUT: glucose transporter
GM-CSF: granulocyte-macrophage colony-stimulating factor
GPX4: glutathione peroxidase 4
GSDMD: gasdermin D
HCC: hepatocellular carcinoma
HFD: high-fat diet
HSC: hepatic stellate cell
HSL: hormone-sensitive lipase
IFN-γ: interferon gamma
Ig: immunoglobulin
IHH: Indian hedgehog
IL: interleukin
ILC: innate lymphoid cell
INF-γ: Interferon gamma
iNKT: invariant natural killer T cell
IR: insulin receptor
JAK: Janus kinase
KCs: Kupffer cells
LAM: lipid-associated macrophage
LIGHT/TNFSF14: tumor necrosis factor superfamily 14
LPS: lipopolysaccharide
LSEC: liver sinusoidal endothelial cell
LTi: lymphoid tissue inducer
MAG: monoacylglycerol
MAIT: mucosal-associated invariant T cell
MASH: metabolic dysfunction-associated steatohepatitis
MASLD: metabolic dysfunction-associated steatotic liver disease
MCD: methionine- and choline-deficient
MD2: Myeloid Differentiation Factor-2
MDM: monocyte-derived macrophage
MGL: monoacylglycerol lipase
MHC: major histocompatibility complex
miR/miRNA: microRNA
MMP: matrix metalloproteinase
MR1: MHC class I-related protein 1
mTOR: mammalian target of rapamycin
MyD88: myeloid differentiation primary response protein 88
Mφ: macrophage
NASH: non-alcoholic steatohepatitis
NET: neutrophil extracellular traps
NF-κB: nuclear factor kappa B
NKG2D: natural killer group 2 member D
NKp46: natural killer cell p46-related protein
NKT: natural killer T cell
NLR: Neutrophil-to-Lymphocyte Ratio
NLRP3: nucleotide-binding domain and leucine-rich repeat protein-3
OSE: oxidation-specific epitopes
PDGF: platelet-derived growth factor
PDGFR: platelet-derived growth factor receptor
PD-L1: programmed death ligand 1
PI3K: phosphatidylinositol 3-kinase
PKA: protein kinase A
PPAR: peroxisome proliferator-activated receptor
PRR: pattern recognition receptor
PUFA: polyunsaturated fatty acid
RAE1: retinoic acid early inducible 1 protein
RNA: ribonucleic acid
ROS: reactive oxygen species
SAMs: scar-associated macrophages
SCD1: Stearoyl-CoA desaturase 1
SGLT-2i: -glucose cotransporter 2 inhibitor
SREBP-1c: sterol regulatory element-binding transcription factor 1c
STAT: signal transducer and activator of transcription
TAG: triacylglycerol
TAZ: transcriptional co-activator with PDZ-binding motif
TCR: T cell receptor
TGF-β: transforming growth factor beta
Th: T helper cell
THR-β: thyroid hormone receptor-β
TLR: Toll-like receptor
TNF-R: Tumor necrosis factor receptor
TNF-α: tumor necrosis factor alpha
TRAIL: tumor necrosis factor-related apoptosis-inducing ligand
TRAIL-R: tumor necrosis factor-related apoptosis-inducing ligand receptor
Treg: regulatory T cell
TREM2: triggering receptor expressed on myeloid cells 2
uPAR: urokinase plasminogen activator receptor
UPR: unfolded protein response
VEGF: vascular endothelial growth factor
VLDL: very low-density lipoprotein
XCR1: chemokine receptor expressed on type 1 conventional dendritic cells
